# Limited Clinical Utility of Routine Mycobacterial Cultures for the Management of Diabetic Foot Infections

**DOI:** 10.1093/ofid/ofad558

**Published:** 2023-11-07

**Authors:** Laila M Castellino, Clare McCormick-Baw, Tyler Coye, Peter A Crisologo, John J Hanna, Francesca Lee, Andrew Clark, Michael Fanning, Amanda L Killeen

**Affiliations:** Division of Infectious Diseases and Geographic Medicine, UT Southwestern Medical Center, Dallas, Texas, USA; Parkland Health, Dallas, Texas, USA; Parkland Health, Dallas, Texas, USA; Division of Pathology and Clinical Laboratory Service, UT Southwestern Medical Center, Dallas, Texas, USA; Division of Vascular Surgery and Endovascular Therapy, Baylor College of Medicine, Houston, Texas, USA; Department of Plastic Surgery, UT Southwestern Medical Center, Dallas, Texas, USA; Division of Infectious Diseases and Geographic Medicine, UT Southwestern Medical Center, Dallas, Texas, USA; Department of Internal Medicine and Clinical Informatics, UT Southwestern Clinical Informatics Center, Dallas, Texas, USA; Division of Infectious Diseases and Geographic Medicine, UT Southwestern Medical Center, Dallas, Texas, USA; Division of Pathology and Clinical Laboratory Service, UT Southwestern Medical Center, Dallas, Texas, USA; Division of Pathology and Clinical Laboratory Service, UT Southwestern Medical Center, Dallas, Texas, USA; Division of Infectious Diseases and Geographic Medicine, UT Southwestern Medical Center, Dallas, Texas, USA; Department of Plastic Surgery, UT Southwestern Medical Center, Dallas, Texas, USA

**Keywords:** acid-fast bacillus cultures, diabetic foot infection, nontuberculous mycobacteria

## Abstract

Mycobacterial infections of the foot and ankle are uncommon. In a cohort of 2340 patients with diabetic foot infection (DFI) in a region with increased prevalence of mycobacterial disease, we identified no clinically significant positive cultures over a 3-year period. Routine mycobacterial culture of DFIs is of limited clinical utility.

Diabetes and associated complications, including infection, are a significant burden on United States (US) healthcare infrastructure, impacting an estimated 11.3% and 38% of adults with diabetes and prediabetes, respectively [1]. Hospital discharge data in 2018 revealed that 6.1 per 1000 adults with diabetes required lower extremity amputation. Total direct estimated costs of diagnosed diabetes increased from $188 billion in 2012 to $237 billion in 2017 (2017 dollars) [2]. Diabetic foot infections (DFIs) are typically polymicrobial, primarily involving *Staphylococcus aureus*, followed by gram-negative and anaerobic bacteria [3]. By contrast, mycobacterial infections of the foot and ankle are rare, described only in case reports [4, 5].

Mycobacterial disease is caused by members of the *Mycobacterium tuberculosis* complex (MTb) and nontuberculous mycobacteria (NTM). MTb is classically acquired through inhalation followed by hematogenous dissemination to bone and other tissues. Conversely, musculoskeletal NTM infections are caused by a heterogeneous group of organisms often acquired through traumatic or percutaneous inoculation with occasional hematogenous involvement, especially in immunocompromised patients. Risk factors for mycobacterial infection include genetic predisposition and acquired immunodeficiency such as human immunodeficiency virus (HIV), malignancy, diabetes, malnutrition, chronic renal disease, and advanced age [6]. Non-US birth is a defining risk factor for >70% of tuberculosis (TB) cases diagnosed in the US, while clinical and social risk factors such as immunocompromise, homelessness, substance use, or incarceration are risk factors among US-born persons [7]. In 2020, Dallas County reported 4.02 TB cases per 100 000 population [8], which was twice the overall US rate of 2.2 TB cases per 100 000 population. The incidence of NTM infections is increasing among both immunocompromised and immunocompetent patients [9, 10] with marked geographic variation. Texas has the third-highest number of cases of pulmonary NTM infections per 100 000 population [11].

Mycobacterial infections are diagnostically challenging, requiring prolonged incubation with specialized reagents and media, necessitating increased turnaround time and cost. Treatment involves a combination of surgery and complex antibiotic regimens for several months, exposing patients to potential antibiotic-related adverse effects. Early and accurate diagnosis of mycobacterial bone and soft tissue infections is important for optimal outcomes with minimal antimicrobial exposure. There is no consensus guidance on the utility of routine acid-fast bacilli (AFB) cultures in the management of DFI or orthopedic infections in general, though these cultures are frequently obtained in clinical practice. Given the time and expense involved in performing these cultures, we reviewed our experience as a quality improvement initiative.

The goal of this study was to examine the utility of routine cultures for AFB among patients with DFI at 2 institutions that serve a large population with risk factors for TB and NTM infections.

## METHODS

### Setting

Parkland Health (PH) is a 900-bed public safety-net hospital serving many patients with risk factors for TB including immigrants from TB-endemic countries, incarcerated individuals, and persons experiencing homelessness. PH is the largest single source of referrals for TB to Dallas County Health and Human Services (personal communication, Gary Woo, MD). As the largest provider of HIV care in Dallas, PH has a large population of immunocompromised patients, including patients with transplants, cancer, and other immunocompromising conditions. William P. Clements Jr University Hospital (CUH) is an 880-bed academic medical center with the largest transplant program in North Texas. CUH serves large transplant and cancer populations, in addition to those with diabetes and other immunocompromising conditions [12]. Both institutions have active diabetic limb salvage teams with surgeons from podiatry and vascular surgery, and ad hoc involvement of infectious diseases and endocrinology.

### Study Design

A retrospective chart review of patients undergoing surgery for DFI between 1 September 2019 and 31 December 2022 at PH and CUH was performed. Inclusion criteria were patients ≥18 years, who underwent surgery by the diabetic limb salvage team, with bacterial and/or mycobacterial cultures sent from surgical specimens, including swabs, soft tissue, and bone. We queried the electronic health record (EHR) (Epic, Verona, Wisconsin) to identify patients who met inclusion criteria, and reviewed charts of all patients with positive mycobacterial cultures to determine their treatment and outcome at last known follow-up. Patients were classified as having diabetes as defined by the American Diabetes Association [13] and moderate to severe DFI as defined by the International Working Group on the Diabetic Foot and the Infectious Diseases Society of America [14, 15]. Glycated hemoglobin (HbA1c) was reported based on the last value available in the EHR during the study period. Statistical analyses were performed using R Statistical software (version 4.3.1 R Foundation for Statistical Computing, Vienna, Austria) and results were summarized using descriptive statistics. AFB culture cost ranged from $78 to $358 depending upon the extent of laboratory workup and susceptibility testing (if required) using pricing from a national reference laboratory.

The study was approved by the University of Texas Southwestern Institutional Review Board and Parkland Research Review Committee.

## RESULTS

Between both hospitals, 2340 patients met inclusion criteria. Of these, 2280 (97%) had bacterial cultures performed, with bacterial organisms identified in 1942 cases (83%). A total of 1877 AFB smears and cultures were completed on 1449 (62%) patients, with a median of 2 AFB cultures (interquartile range, 1–3) sent per patient, of which 248 specimens were from bone.

Patients with AFB cultures performed (Table 1) were predominantly male (72%), with an average age of 57 years. Forty-six percent self-identified as ethnically Hispanic, and 70% had HbA1c >7%. At PH, 58% of patients identified as Hispanic, and at CUH 22% identified as Hispanic, reflective of general patient demographics at both hospitals. Twenty percent of PH patients had well-controlled diabetes (HbA1c <7%) versus 50% of CUH patients.

**Table 1.** Epidemiological Characteristics of Patients With Acid-Fast Bacillus Cultures

Characteristic	Overall (N = 1449)	CUH (n = 487)	PH (n = 962)
Age, y, mean (SD)	57.61 (13.03)	62.53 (14.36)	55.12 (11.53)
Female sex	404 (28%)	174 (36%)	230 (24%)
Hispanic ethnicity	671 (46%)	108 (22%)	563 (59%)
Race			
Asian	14 (1.0%)	7 (1.5%)	7 (0.8%)
Black	335 (24%)	123 (26%)	212 (24%)
Other	26 (1.9%)	22 (4.6%)	4 (0.4%)
Unknown	112 (7.7%)	34 (7.0%)	72 (7.5%)
White	962 (70%)	301 (62%)	661 (74%)
Glycated hemoglobin			
≤7%	431 (30%)	242 (50%)	189 (20%)
>7%	1018 (70%)	245 (50%)	773 (80%)
Positive aerobic cultures	1102 (76%)	323 (66%)	779 (81%)
Positive anaerobic cultures	596 (41%)	155 (32%)	441 (46%)
Positive fungal cultures	125 (9%)	41 (8%)	84 (9%)
Positive AFB cultures	(0.13%)	1 (0.20%)	1 (0.10%)
Total No. of AFB cultures per patient, median (IQR)	2.00 (1.00–3.00)	2.00 (1.00–3.00)	1.00 (1.00–2.00)

Data are presented as No. (%) unless otherwise indicated.

Abbreviations: AFB, acid-fast bacillus; CUH, William P. Clements Jr. University Hospital; IQR, interquartile range; PH, Parkland Health; SD, standard deviation.

Of the 1877 AFB cultures completed, 2 were positive for AFB. One culture obtained from soft tissue was positive for *Mycobacterium avium*/*intracellulare* (MAI). The patient was a 51-year-old woman with diabetes and end-stage renal disease (ESRD) on peritoneal dialysis, with a left foot wound that probed to bone, and underwent an incision and drainage with fifth metatarsal ray amputation. Deep soft tissue cultures concomitantly grew *Staphylococcus aureus*. Histopathology revealed acute inflammation of soft tissue and bone consistent with osteomyelitis; no granulomatous changes were noted. Based on clinical and histopathological findings, the recovered MAI was determined to be a contaminant not warranting treatment. The patient received intravenous cefazolin followed by oral doxycycline for 6 weeks. At a follow-up visit 9 months later, the left foot incision was well healed.

A second positive culture for *Mycobacterium abscessus* was noted. The patient was a 74-year-old man with diabetes, ESRD on hemodialysis, peripheral vascular disease, and multiple gangrenous digits requiring a left transmetatarsal amputation (TMA) with wound dehiscence requiring subsequent revision TMA. Bone cultures were positive for *Pseudomona*s *aeruginosa* and *Enterobacter cloacae*, and 1 of 2 AFB cultures grew *M. abscessus*. Histopathology from the proximal margin of all 5 metatarsals was negative for osteomyelitis, and the patient did not receive any *M. abscessus*–directed therapy. At last known follow-up 14 months later, the TMA was well healed with no signs of infection.

Based on a cost of $78 per negative AFB culture, and $358 per positive AFB culture, we calculated that $146 966 of healthcare expenditure could have been avoided, which includes an estimated 40 minutes per culture spent processing tissue specimens and reviewing slides and plates evaluating for AFB. This totals 1251 laboratory hours spent by medical laboratory scientists to process these cultures.

## DISCUSSION

We retrospectively interrogated the clinical impact of routine AFB cultures of DFI at 2 institutions over 3 years. Although we noted 2 cultures with NTM, neither was clinically significant nor specifically treated. At follow-up, both patients with positive AFB cultures had well-healed wounds despite no NTM-specific therapy, adding to the growing body of literature that routine AFB cultures are not warranted in musculoskeletal infections, including DFI [16, 17]. There is a significant financial and labor cost to the institution with respect to personnel time required to perform AFB stains and cultures, which are incubated for 6 weeks. Patients and payors also shoulder financial burden for these cultures, which ultimately do not alter clinical management.

A limitation of our study is that our population may not be representative of other geographic areas. Differences in microbiological etiology of DFI have been described between temperate and nontemperate parts of the world [18]. Hence our findings might not be generalizable, especially in areas of the world where TB is endemic. Additionally, the number of bone cultures sent for AFB testing is an underestimate, as they are sometimes labeled as a swab with a comment stating this is from bone, which is not captured in our review. That said, in a large cohort of patients with DFI at 2 academic urban medical centers in Texas, where the general rate of TB and NTM disease prevalence is higher than other areas in the US, we did not find any clinically useful results from routine AFB cultures. Additionally, there is potential for harm from unnecessary exposure to antibiotic therapy, due to AFB culture contamination. Our findings support review and restructuring of intraoperative laboratory ordering processes as a diagnostic stewardship intervention, to avoid routine AFB cultures for DFI, limit potentially unnecessary antibiotic exposure, and decrease the financial burden from a labor-intensive assay with limited clinical impact.

## Notes

***Acknowledgments.*** We thank Dr. Gary Woo of Dallas County Health and Human Services for his insights on the epidemiology of tuberculosis in Dallas County.

***Patient consent.*** The design of this work was approved by the University of Texas Southwestern Institutional Review Board and the Parkland Research Review Committee. Individual patient consent was not required due to the retrospective nature of the study.

## References

[1] Centers for Disease Control and Prevention . National diabetes statistics report. 2022. Available at: https://www.cdc.gov/diabetes/data/statistics-report/index.html. Accessed 18 June 2023.

[2] American Diabetes Association . Economic costs of diabetes in the U.S. in 2017. Diabetes Care 2018; 41:917–28. 29567642 10.2337/dci18-0007 PMC5911784

[3] Macdonald KE, Boeckh S, Stacey HJ, Jones JD. The microbiology of diabetic foot infections: a meta-analysis. BMC Infect Dis 2021; 21:770. 34372789 10.1186/s12879-021-06516-7 PMC8351150

[4] Hingorani A, Ascher E, Hingorani A. The great masquerader—TB osteoarthritis. Ann Vasc Surg 2022; 78:377.e1–3. 10.1016/j.avsg.2021.06.030 34481885

[5] Lazzarini L, Amina S, Wang J, Calhoun JH, Mader JT. *Mycobacterium tuberculosis* and *Mycobacterium fortuitum* osteomyelitis of the foot and septic arthritis of the ankle in an immunocompetent patient. Eur J Clin Microbiol Infect Dis 2002; 21:468–70. 12111605 10.1007/s10096-002-0742-0

[6] Hogan JI, Hurtado RM, Nelson SB. Mycobacterial musculoskeletal infections. Thorac Surg Clin 2019; 29:85–94. 30454925 10.1016/j.thorsurg.2018.09.007

[7] Langer AJ, Navin TR, Winston CA, LoBue P. Epidemiology of tuberculosis in the United States. Clin Chest Med 2019; 40:693–702. 31731978 10.1016/j.ccm.2019.07.001 PMC6878887

[8] Texas Department of State Health Services . Texas health data: tuberculosis in Texa. Availabe at: https://healthdata.dshs.texas.gov/dashboard/diseases/tuberculosis-in-texas. Accessed 29 May 2023.

[9] Lopez M, Croley J, Murphy KD. Atypical mycobacterial infections of the upper extremity: becoming more atypical? Hand (N Y) 2017; 12:188–92. 28344532 10.1177/1558944716642764 PMC5349403

[10] Wentworth AB, Drage LA, Wengenack NL, Wilson JW, Lohse CM. Increased incidence of cutaneous nontuberculous mycobacterial infection, 1980 to 2009: a population-based study. Mayo Clin Proc 2013; 88:38–45. 23218797 10.1016/j.mayocp.2012.06.029 PMC3690780

[11] Strollo SE, Adjemian J, Adjemian MK, Prevots DR. The burden of pulmonary nontuberculous mycobacterial disease in the United States. Ann Am Thorac Soc 2015; 12:1458–64. 26214350 10.1513/AnnalsATS.201503-173OC PMC4627421

[12] Scientific Registry of Transplant Recipients . January 2023 data. Available at:. https://www.srtr.org/reports/program-specific-reports. Accessed 5 July 2023.

[13] ElSayed NA, Aleppo G, Aroda VR, et al 2. Classification and diagnosis of diabetes: standards of care in diabetes—2023. Diabetes Care 2023; 46(Suppl 1):S19–40.36507649

[14] Lipsky BA, Senneville E, Abbas ZG, et al Guidelines on the diagnosis and treatment of foot infection in persons with diabetes (IWGDF 2019 update). Diabetes Metab Res Rev 2020; 36(Suppl 1):e3280. 32176444 10.1002/dmrr.3280

[15] Lipsky BA, Berendt AR, Cornia PB, et al 2012 Infectious Diseases Society of America clinical practice guideline for the diagnosis and treatment of diabetic foot infections. Clin Infect Dis 2012; 54:e132–73. 22619242 10.1093/cid/cis346

[16] Tai DBG, Wengenack NL, Patel R, Berbari EF, Abdel MP, Tande AJ. Fungal and mycobacterial cultures should not be routinely obtained for diagnostic work-up of patients with suspected periprosthetic joint infections. Bone Joint J 2022; 104-B:53–8. 34969277 10.1302/0301-620X.104B1.BJJ-2021-0876.R1

[17] Sanderford J, Krumrey J, Campaigniac E, Lin J, Tsai P, Pipitone O. The utility of acid-fast bacillus (AFB) and fungal cultures in orthopaedic infections. Cureus 2022; 14:e26639. 35949753 10.7759/cureus.26639 PMC9357251

[18] Hatipoglu M, Mutluoglu M, Turhan V, et al Causative pathogens and antibiotic resistance in diabetic foot infections: a prospective multi-center study. J Diabetes Complications 2016; 30:910–6. 26965794 10.1016/j.jdiacomp.2016.02.013

